# The role of endothelial junctions in the regulation of the extravasation of tumor cells. A historical reappraisal

**DOI:** 10.3389/fonc.2024.1415601

**Published:** 2024-07-05

**Authors:** Domenico Ribatti

**Affiliations:** Department of Translational Biomedicine and Neuroscience, University of Bari Medical School, Bari, Italy

**Keywords:** endothelial junctions, exosomes, extravasation, metastasis, tumor growth

## Abstract

Endothelial cells lining the vessel wall are connected by adherent, tight and gap junctions. Adherent junctions are common to all endothelial cells, whereas tight and gap junctions graduate within different vascular segments. Endothelial cell-cell junctions sustain vascular homeostasis and to control the transendothelial migration of inflammatory cells. Tumor cells need to weaken endothelial cell-cell junctions to penetrate the endothelial barrier and transendothelial migration and metastasis of tumor cells are tightly controlled by endothelial cell-cell junctions.

## Interendothelial junctions

**Figure 1:**
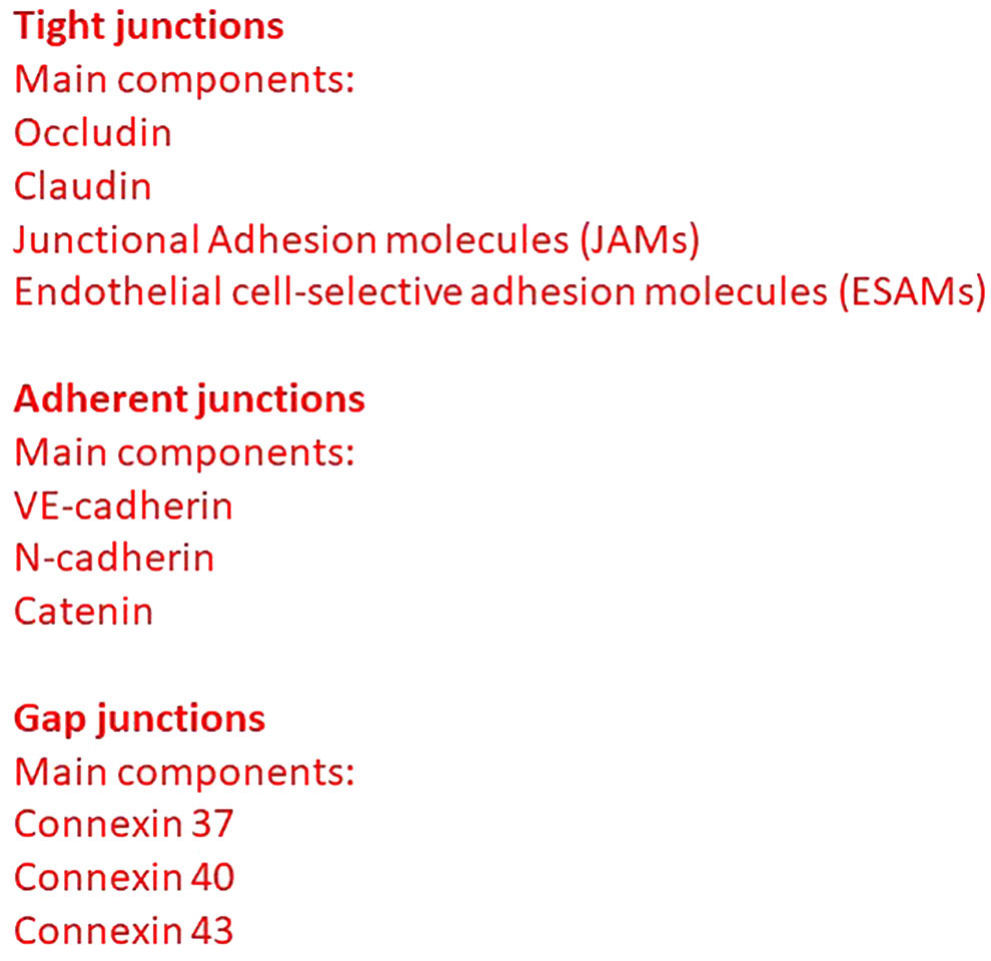
Different types of junctions present in the vascular endothelial cells.

Endothelial cells lining the vessel wall are connected by adherent, tight and gap junctions (1). Adherent junctions are common to all endothelial cells, whereas tight and gap junctions graduate within different vascular segments (**Figure 1**) (2).

Tight junctions (also termed *zona occludens*) are highly impermeable junctions in the apical side of interendothelial membrane, consisting of claudins, occludin, junctional adhesion molecules (JAMs), endothelial cell-selective adhesion molecules (ESAMs), and other adhesion molecules (3). Occludin establishes a barrier function through a relationship with zona occludens 1 (ZO-1), which in turn interacts with α-catenin and actin in the cytoskeleton. JAM-A, JAM-B, and JAM-C are components of the immunoglobulin superfamily. JAM-A and JAM-C are also expressed in the epithelia and the leukocytes, whereas JAM-B is present also in Sertoli cells. Tight junctions intervene also in the regulation of cellular permeability and the transduction of cellular signaling. Adherent junctions (also termed *zona adherens*) provide the structural base for interendothelial mechanical stability and maintenance. Vascular endothelial (VE)-cadherin is present in adherent junctions and is essential for the maintenance of endothelial barrier integrity; in fact, VE-cadherin blocking antibody is responsible for an increase in vascular permeability (1). VE-cadherin interacts with cytosolic molecules α catenin, β catenin, plakoglobin (or γ catenin), and p120, and stabilizes tight junctions promoting claudin-5 expression (4).

Vascular endothelial growth factor (VEGF) and transforming growth factor beta 1 (TGFβ1) secreted by tumor cells induce the endothelial cell junction opening of the endothelial cell junctions interfering with the VE-cadherin and β catenin (5). Moreover, tumor cells also secrete angiopoietin-like 4 (ANGPTL4) and C-C-chemokine ligand 2 (CCL2), which antagonize endothelial cell tight junctions, promoting tumor cells extravasation (6). Finally, C-X-C chemokine ligand-4 secreted by endothelial cells promotes cancer cells’ transendothelial migration through interaction with C-X-C chemokine receptor type 4 and 7 (CXCR4 and CXCR7) expressed by tumor cells (7).

Connexins 37, 40, and 43, in the form of hexamers, intervene in the structure of endothelial gap junctions giving rise to hemichannels joining their counterparts in adjacent cells and controlling vascular permeability (8).

## Leukocyte extravasation

Post-capillary venules are involved in the control of extravasation of leukocytes during inflammatory processes. Leukocytes attach, roll, adhere, and transmigrate through the altered interendothelial junctions. Rolling is mediated by interactions between platelet (P)- and endothelial (E)-selectins, and leukocyte carbohydrate-based ligands, whereas in adhesion are involved the interactions between leukocyte integrins and endothelial intercellular adhesion molecule 1 (I-CAM-1) and vascular cellular adhesion molecule-1 (V-CAM-1) (9). Leukocyte-endothelial interactions may also occur in large veins, capillaries, and arterioles. While leukocytes extravasate in the post-capillary venules of the skin, muscle, and mesentery, in other organs, such as the lung and liver, leukocytes pass across the microvasculature.

High endothelial venules (HEVs), characterized by plumb or cuboidal endothelial cells, are involved in the recirculation of lymphocytes between blood and lymph nodes (10). In HEVs, the process of transmigration of leukocytes involves the binding of lymphocyte (L)-selectin to peripheral lymph node addressin (PNAd) and mucosal addressin cell adhesion molecule 1 (MadCAM-1) expressed by endothelial cells, and the interaction between leukocyte integrins and endothelial ICAM-1, ICAM-2, and MadCAM-1 (11).

Leukocytes may transmigrate between (the paracellular route) or through (the transcellular route) endothelial cells. Paracellular extravasation occurs through passage across intercellular gaps between adjacent endothelial cells (12). Platelet/endothelial cell adhesion molecule-1 (PECAM-1)/CD31, JAM-1, and CD99 are involved in transendothelial cell migration (9). Blockade of PECAM-1/CD31 inhibits the transmigration of neutrophils, monocytes, and natural killer (NK cells). Otherwise, this receptor is dispensable for the transmigration of lymphocytes (13). Activated endothelial cells express chemokines responsible for integrin activation on the leukocyte surface. The binding of the integrin leukocyte function-associated antigen-1 (LFA-1) and very late activated antigen-4 (VLA-4) to the respective ligands ICAM-1 and VCAM-1 on endothelial cells regulates leukocyte adhesion and spreading (14). Tumor necrosis factor alpha (TNF-α) and interleukin 1 beta (IL-1β) activate endothelial cells, inducing the release of CCL2 and CCL7 that, in turn, recruit NK cells and induce the expression of ICAM-1 and VCAM-1 (15).

## Junctional complexes in tumor endothelial cells

Tumor endothelial cells are characterized by altered morphologic and genetic phenotype when compared to normal endothelial cells. Tumor endothelial cells are primarily involved in priming, activation, and down-regulation of effector immune cells, and in this context, they directly impact on anti-cancer immune responses.

VE-cadherin breakdown promotes hematogenic metastasis and constitutes a potential therapeutic target (16). Neuronal cadherin (N-cadherin) expressed by tumor and endothelial cells is responsible for the attachment of cancer cells to the endothelium (17). Knocking down of N-cadherin or anti-sense RNA-mediated repression of N-cadherin reduces the interactions of melanoma cells with the endothelium, and transendothelial migration (17, 18).

Down-regulation of tight junction is involved in uncontrolled tumor cell growth, detachment, and invasion of cancer cells and hence successful penetration of the endothelium by cancer cells. The level of expression of ZO-1 was reduced or lost in 69% of breast cancer as compared to normal tissue (19). In infiltrating ductal carcinomas, a reduction in ZO-1 immunohistochemical expression has been demonstrated in 93% of poorly differentiated tumors. Moreover, the proteins S100A4 and S100A8/A9 overexpressed in different types of cancer downregulate tight junction protein occludin (20). Bladder cancer cells release angiomodulin, which interacts with αvβ3 integrin, and favors the formation of actin stress fibers leading to weakened intercellular connections mediated by VE-cadherin (21). In glioma stem-like cells, semaphorin 3A binds to Neuropilin 1-plexin A1 (NRP-1-plxA1) complex and activates Src, facilitating the relocation of VE-cadherin (22).

## Tumor endothelial cell permeability, leukocyte trafficking, and extracellular matrix

**Figure 2:**
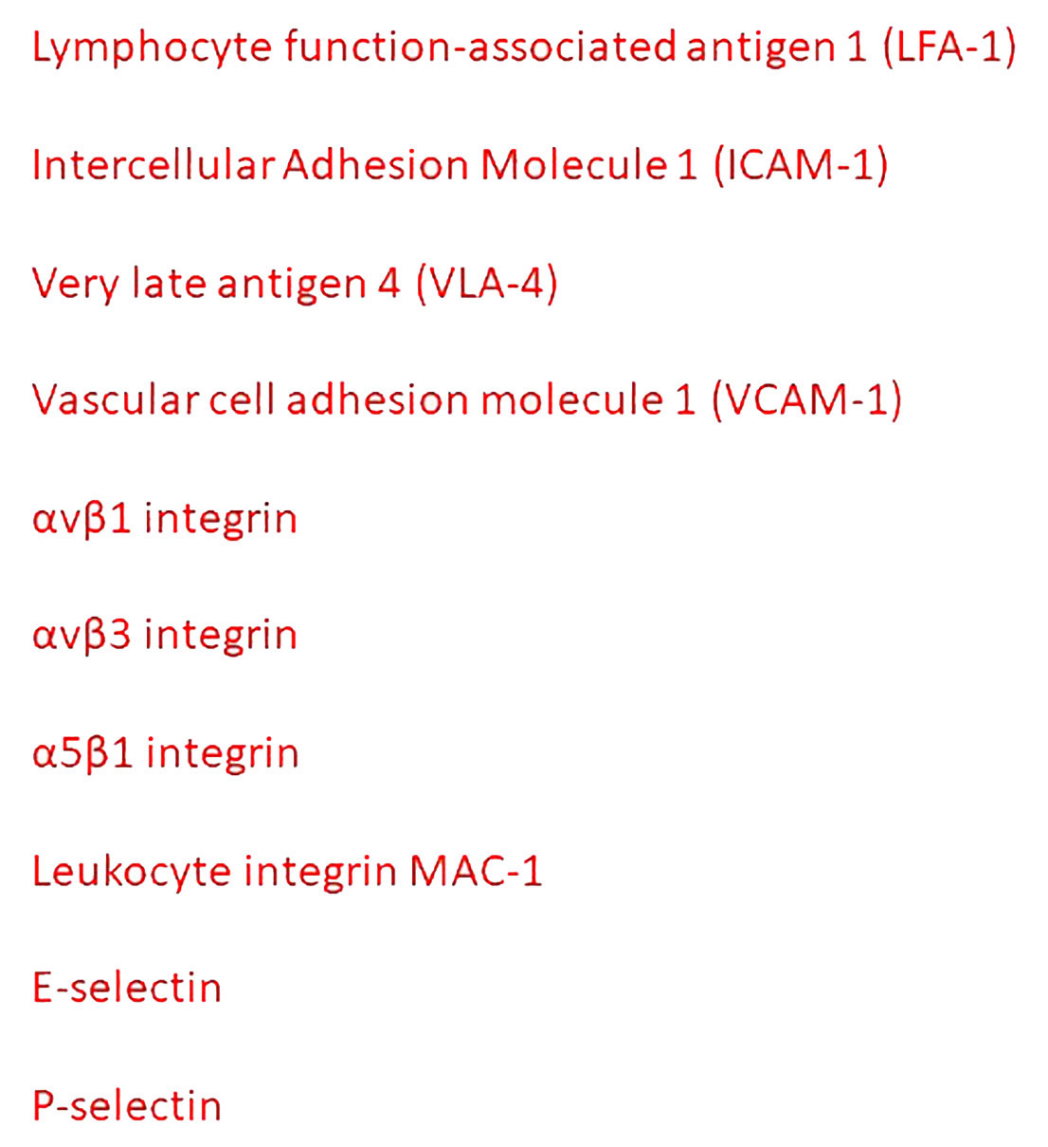
Principal mediators of the interactions between inflammatory cells and tumor endothelial cells.

In tumor chronic inflammation, an increase in vascular permeability can persist over time (**Figure 2**) (23). Neutrophils interact with circulating tumor cells (24), and modify the behavior of the tumor cells, conferring a more aggressive phenotype. Lymphocyte function adhesion molecule-1 (LFA-1) is responsible for the initial capture of neutrophils, whereas the interaction between tumor ICAM-1 and neutrophil MAC-1 is involved in maintaining clusters of neutrophils (25). Neutrophils produce MAC-1 and LFA-1 during extravasation, which bind to ICAM-1 expressed on the endothelium (25). In melanoma, IL-8 produced by cancer tumor cells attracts neutrophils, that promotes by induction of MAC-1 and ICAM-1 the interaction between cancer tumor cells and neutrophil clusters, and the endothelium (26). Tumor endothelial cells altered glycosylation of ICAM-1, VCAM-1, and PECAM, and lectins, promoting tumor progression and metastasis, and modifying the adhesive properties of endothelial cells (27). Blocking the interactions between circulating tumor cells and endothelium is a potential target to inhibit metastasis.

Tumor-associated macrophages (TAMs) modulate vascular permeability through VEGF-promoted down-regulation of endothelial ZO-1 and VE-cadherin, and VLA-4-mediated disruption of endothelial VCAM-1 (28, 29).

Tumor endothelial cells increase the expression of the common lymphatic endothelial and vascular endothelial receptor-1 (CLEVER-1), expressed on lymphatic endothelial cells, sinusoidal endothelial cells in the liver and spleen, and HEVs. CLEVER-1 favors the selective transmigration of Tregs and type II macrophages from the blood into the tumor (30).

Alpha 4 integrin is expressed in many different human tumors, including melanoma and sarcoma. The α4β1 integrin expressed by cancer cells may act as an alternative ligand for VCAM-1 to mediate the adhesion of cancer cells to the endothelium (31). The interaction between αvβ1 or αvβ3 integrins and neuronal cell adhesion molecule is also involved in the interaction between tumor and endothelial cells (32). Leukocytes expressing αLβ2 integrins link tumor cells and endothelial cells expressing ICAM-1 (33). Epithelial tumor cells synthesize ANGPTL4 and bind to endothelial integrin α5β1, claudin-5, and VE-cadherin, weakening the cellular junctions (34). ANGPTL4 induces retraction of endothelial cells from one another, leaving gaps in the capillary walls that facilitate extravasation as indicated by the dissolution of the ZO-1 containing tight junctions between adjacent cells. STING activation synergizes with VEGF receptor 2 (VEGFR-2) blockade, leading to normalization of the tumor vasculature, upregulation of VCAM-1 and ICAM-1, and regression of immunotherapy-resistant tumors (35).

E-and P-selectins facilitate the adhesion of cancer cells to the endothelium (36). Cancer cells express different ligands specific for endothelial selectins, including hematopoietic cell E-/L-selectins ligand (HCELL), carcinoembryonic antigen (CEA), and P-selectin glycoprotein ligand 1 (PSGL1) (36). The binding between P-selectin expressed by platelets and selectin ligands on cancer cells bridges tumor cells and platelets, favoring the adhesion of tumor cells to endothelial cells (36). E- and P-selectins are also involved in colon and breast cancer metastasis (37, 38). Metastases are reduced by 50% when small cell lung cancer cells are xenografted in E- and P-selectin-deficient mice (39). Administration of GMI-1271, a small molecule E-selectin antagonist, reduces cancer cell attachment to the endothelium (40). In patients with metastatic melanoma, the combination of bevacizumab with anti-cytotoxic T-lymphocyte associated protein 4 (CTLA-4) monoclonal antibody increases the expression of E-selectin, ICAM-1, and VCAM-1, and enhances T cell recruitment in the tumor microenvironment and improves the clinical outcome (41).

## Exosomes and angiopellosis

Exosomes are small vesicles with a diameter of 30–200 nm, containing nucleic acids, proteins, and other substances. Different pro-angiogenic factors including VEGF, TGFβ, fibroblast growth factor 2 (FGF-2), IL-6, and IL-8, are present in exosomes derived from tumor cells. Moreover, tumor cell exosomes can be uptake by endothelial cells and can break down vascular integrity (42). In colorectal cancer, the cancer cell-derived exosomes contain miRNA that downregulates ZO-1, destroying the endothelial barrier (43). TAMs-derived exosomes containing miR-23a, miR-155, and miR-221 induced vascular leakiness in hepatocellular carcinoma (44). Finally, cancer-derived exosomes are the main driving force for metastasis niche formation.

Cancer cell clusters that possess a higher metastatic potential can extravasate using an alternative mechanism named angiopellosis, consisting of active remodeling of endothelial cells to cover the extravasating cells and then push these cells out of the blood vessels (45). The endothelial cells extend protrusions and actively remodel themselves around the exiting cells. The exiting cell will then be either actively “pushed” from the inside of the blood vessel, or the vascular cells will remodel around the cell so that the cell no longer remains inside the vessel.

## Discussion

The reorganization of endothelial cell junctions and cytoskeleton allows cancer cells to pass through. Tumor cells induce rapid endothelial cell dissociation, leading to the loss of VE-cadherin expression and changes in vinculin distribution and organization. The hyperpermeable nature of the tumor microcirculation is well established and depends on altered interendothelial junctions, transendothelial channels, fenestrations, and vescicular vacuolar organelles. In this context, it has been speculated that large therapeutic agents could be selectively delivered to the tumors. Widened interendothelial junctions have been described in tumor vessels (46). In the brain vasculature, the modulation of claudin-5 and angulin-1 enables the blood-brain barrier permeability of molecules of molecular weight less than 1 and 5.3 kDa, respectively (47). Moreover, the co-regulation of claudin-5 and occludin increased the permeability of molecules as large as 4 kDa (48), indicating that targeted tight junction components are important in controlling the size of molecules that pass through the blood-brain barrier in tumors. Viruses can be utilized as tools to induce the opening of tight junctions and enhance the permeability of the blood-brain barrier (49).

Cancer metastasis is a complex event involving cancer cells and the endothelial junctional complexes (32). Adequate knowledge of the interactions as well as the role individual role played by these junctions could serve as a target for the treatment of metastasis. Agents that inhibit the effects of cytokines and growth factors such as TNFα, TGFβ, VEGF, and HGF, all involved in the increase of vascular permeability, decrease transepithelial/endothelial resistance and increase paracellular permeability, could be a useful tool against cancer metastasis. Drugs that neutralize VEGF, inhibit VEGFR-2 or activate Tie2 are effective in reducing vascular permeability and leakage.

## Author contributions

DR: Writing – original draft, Writing – review & editing.
